# Divergent effects of genetic and pharmacological inhibition of Nox2 NADPH oxidase on insulin resistance-related vascular damage

**DOI:** 10.1152/ajpcell.00389.2019

**Published:** 2020-05-13

**Authors:** Azhar Maqbool, Nicole T. Watt, Natalie Haywood, Hema Viswambharan, Anna Skromna, Natalia Makava, Asjad Visnagri, Heba M. Shawer, Katherine Bridge, Shovkat K. Muminov, Kathryn Griffin, David J. Beech, Stephen B. Wheatcroft, Karen E. Porter, Katie J. Simmons, Piruthivi Sukumar, Ajay M. Shah, Richard M. Cubbon, Mark T. Kearney, Nadira Y. Yuldasheva

**Affiliations:** ^1^Leeds Institute for Cardiovascular and Metabolic Medicine, University of Leeds, Leeds, United Kingdom; ^2^Tashkent Pediatric Medical Institute, Tashkent, Uzbekistan; ^3^British Heart Foundation, Centre of Research Excellence, King’s College London, London, United Kingdom

**Keywords:** atherosclerosis, insulin resistance, Nox2

## Abstract

Insulin resistance leads to excessive endothelial cell (EC) superoxide generation and accelerated atherosclerosis. The principal source of superoxide from the insulin-resistant endothelium is the Nox2 isoform of NADPH oxidase. Here we examine the therapeutic potential of Nox2 inhibition on superoxide generation in saphenous vein ECs (SVECs) from patients with advanced atherosclerosis and type 2 diabetes and on vascular function, vascular damage, and lipid deposition in apolipoprotein E-deficient (ApoE^−/−^) mice with EC-specific insulin resistance (ESMIRO). To examine the effect of genetic inhibition of Nox2, ESMIRO mice deficient in ApoE^−/−^ and Nox2 (ESMIRO/ApoE^−/−^/Nox2^−/y^) were generated and compared with ESMIRO/ApoE^−/−^/Nox2^+/y^ littermates. To examine the effect of pharmacological inhibition of Nox2, we administered gp91dstat or scrambled peptide to ESMIRO/ApoE^−/−^ mice. SVECs from diabetic patients had increased expression of Nox2 protein with concomitant increase in superoxide generation, which could be reduced by the Nox2 inhibitor gp91dstat. After 12 wk Western diet, ESMIRO/ApoE^−/−^/Nox2^−/y^ mice had reduced EC superoxide generation and greater aortic relaxation to acetylcholine. ESMIRO/ApoE^−/−^/Nox2^−/y^ mice developed more lipid deposition in the thoraco-abdominal aorta with multiple foci of elastin fragmentation at the level of the aortic sinus and greater expression of intercellular adhesion molecule-1 (ICAM-1). Gp91dstat reduced EC superoxide and lipid deposition in the thoraco-abdominal aorta of ESMIRO/ApoE^−/−^ mice without causing elastin fragmentation or increased ICAM-1 expression. These results demonstrate that insulin resistance is characterized by increased Nox2-derived vascular superoxide. Complete deletion of Nox2 in mice with EC insulin resistance exacerbates, whereas partial pharmacological Nox2 inhibition protects against, insulin resistance-induced vascular damage.

## INTRODUCTION

Insulin resistant type 2 diabetes is a chronic systemic disorder that leads to deleterious changes in the blood vessel wall (26) and premature cardiovascular disease (1). Despite the use of contemporary treatments, individuals suffering from insulin resistant type 2 diabetes have mortality rates from the complications of cardiovascular disease at least three times that of an individual without type 2 diabetes (4). As the global population of humans suffering from type 2 diabetes continues to increase at an alarming rate (11), new therapies and mechanistic understandings addressing insulin resistance-related vascular disease are urgently needed.

One pathophysiological process thought to make a major contribution to type 2 diabetes-related vascular disease is unrestrained generation of cytotoxic concentrations of the free radical superoxide from the endothelial lining of the arterial wall (2, 9). This so called “oxidative stress” or “endothelial dysfunction” has a range of effects that could accelerate the development of vascular disease (2), principal among which is thought to be oxidative modification of circulating low-density lipoprotein (LDL), which leads to an adhesion molecule and inflammatory cell-mediated change in the architecture of the arterial wall facilitating subintimal deposition of LDL cholesterol (31).

Previously, we have demonstrated that the principal enzymatic source of superoxide from the endothelium in insulin resistance is the Nox2 isoform of nicotinamide adenine dinucleotide phosphate oxidase (Nox2) (5, 6, 30, 32, 33). We also showed that inhibition of Nox2 using pharmacological or genetic approaches can reduce superoxide generation and improve endothelial function in mice with endothelium-specific or whole body insulin resistance (30).

Here we examine the therapeutic potential of inhibiting Nox2 on oxidative stress, vascular damage, and arterial lipid deposition in atherosclerosis-prone mice with endothelial cell-specific insulin resistance.

We report the following key findings. First, endothelial cells from humans with advanced atherosclerosis and diabetes have increased expression of Nox2 and increased superoxide generation, which is reduced by specific inhibition of Nox2. Second, mice with endothelial-specific insulin resistance that were also deficient in Nox2 and on an apolipoprotein E (ApoE)-deficient background develop increased lipid deposition in the thoraco-abdominal aorta, significant elastin fragmentation at the level of the aortic sinus, and increased expression of the adhesion molecule ICAM-1, despite reduced superoxide generation from endothelial cells and enhanced endothelial-dependent vasorelaxation. Third, treating mice, which are both deficient in ApoE and display endothelium-specific insulin resistance, with the Nox2-specific inhibitor gp91dstat reduced superoxide generation and deposition of lipid in the thoraco-abdominal aorta without elastin fragmentation or increasing ICAM-1 expression.

## MATERIAL AND METHODS

### 

#### Isolation of primary saphenous vein endothelial cells from patients with advanced atherosclerosis.

Patients undergoing aortocoronary artery bypass surgery were recruited from the Department of Cardiac Surgery at Leeds Teaching Hospitals. Primary saphenous vein (SV) endothelial cells (SVECs) were isolated from segments of human SV, obtained as previously described (18). Ethical approval was granted by the local Research Ethics Committee (Ref. No. CA01/040). SVECs were grown in EBM-2 growth medium supplemented with an EGM-2 bullet kit (Lonza) and used up to passage 3.

#### Generation of mice with EC-specific insulin resistance on a proatherogenic background.

To examine the effect of genetic and pharmacological inhibition of Nox2 on insulin resistance-related vascular disease, we generated mice with EC-specific insulin resistance due to expression of mutant human insulin receptors under the Tie2 promoter-enhancer (ESMIRO), which were also deficient in apolipoprotein E [ApoE^−/−^ (8)] and Nox2 (Nox2^−/y^). See Supplemental Fig. S1 (see https://doi.org/10.6084/m9.figshare.11967792) for the breeding and genotyping strategy. To determine the transgenic status during breeding, mice were genotyped using ear notch DNA. Three genotyping reactions (ApoE, ESMIRO, and Nox2) were established details of which are provided in Supplemental Fig. S2 and in Supplemental Tables S1–S3.

Mice were maintained in a temperature- and humidity-controlled environment on a 12-h light-dark cycle. Male mice and their littermate controls were studied in all experiments, which were conducted in accordance with accepted standards of humane animal care under United Kingdom Home Office Project licenses no. 40/3523 and P144DD0D6.

#### Treatment with the Nox2-specific inhibitor gp91dstat.

To examine the effect of pharmacological inhibition of Nox2 on the development of insulin resistance-related vascular disease, we performed chronic treatment studies using the Nox2-specific inhibitory peptide gp91dstat (3, 23). At 8 wk of age, mice were placed on a high fat, high cholesterol, proatherogenic, Western style diet (cat. no. 829100, Dietex). After 4 wk of Western diet, the mice were anesthetized and osmotic minipumps (Alzet 1004) containing 10 mg·kg^−1^·day^−1^ gp91dstat or scrambled peptide were implanted (30, 32). The pumps were replaced after 4 wk and left in place for a further 4 wk.

#### Metabolic testing.

Glucose and insulin tolerance tests were performed by blood sampling after an intraperitoneal injection of glucose (1 mg/g; Sigma-Aldrich, UK) or human recombinant insulin (0.75 unit/kg: Actrapid; Novo Nordisk, Bagsvaerd, Denmark), as previously described (5, 6, 30, 32, 33). Glucose concentrations were determined in whole blood using a portable meter (Roche Diagnostics, Burgess Hill, UK). Plasma insulin concentrations were determined by enzyme-linked immunoassay (Ultrasensitive mouse ELISA; CrystalChem, Downers Grove, IL). Triglycerides and total cholesterol were quantified as described previously (8). Liver function was assessed by measuring blood serum levels of alanine aminotransferase (ALT) and aspartate transaminase (AST) (74707, 74032, Advia Chemistry, Siemens).

#### Arterial blood pressure.

Systolic blood pressure was measured by tail-cuff plethysmography (Kent Scientific, Torrington, UK) as previously described (5, 6, 30).

#### Studies of vasomotor function in aortic rings.

Vasomotor function was assessed in aortic rings as previously described (5, 6, 30, 32, 33). Rings were mounted in an organ bath containing Krebs-Henseleit buffer and equilibrated at a resting tension of 3 *g* for 45 min before the experiments. A cumulative dose response to the constrictor phenylephrine (1 nmol/L to 10 μmol/L) was performed. Relaxation responses to the cumulative addition of acetylcholine (1 nmol/L to 10 µmol/L) and sodium nitroprusside (SNP) (0.1 nmol/L to 1 µmol/L) were performed, and responses are expressed as percent decrement in preconstricted tension.

#### Pulmonary endothelial cell isolation and culture.

Pulmonary endothelial cells (PECs) were isolated by immunoselection with CD146 antibody-coated magnetic beads as previously described and resuspended and plated in MV2 medium (Promocell), supplemented with MV2 supplement, 100 units/mL penicillin, and 100 μg/mL streptomycin (5, 6, 30, 32, 33). The endothelial cell population tested positive for a range of endothelial markers including endothelial nitric oxide synthase (eNOS), Tie2, ve-cadherin, von Willebrand factor (vWF), and CD102 protein. The cells were used from fresh isolates (P0) and were not further passaged.

#### Lucigenin enhanced chemiluminescence assessment of NADPH-dependent superoxide generation.

We used lucigenin enhanced chemiluminescence to measure NAD(P)H-dependent superoxide production in PECs, as previously described (5, 6, 30, 32, 33). All experiments were performed in triplicate and luminescence measured upon addition of a non-redox cycling concentration of lucigenin (5 µM) and NADPH (100 µM), using a VarioSkan 96-well microplate luminometer (Thermo Scientific).

#### Quantification of circulating leukocyte populations.

Heparinized whole venous blood underwent erythrocyte lysis (Pharmalyse, BD Biosciences) before the isolation of peripheral blood mononuclear cells (PBMCs) by centrifugation. After washing and resuspending PBMCs in PBS with 0.5% BSA and 2 mM EDTA, cells were incubated at 4°C with CD16/32 Fc block (130-092-575, Miltenyi Biotec) for 10 min. Anti-CD45-VioBlue (130-110-802, Miltenyi Biotec), anti-CD11b-FITC (130-081-201, Miltenyi Biotec), anti-Ly6G-PE (130-107-913, Miltenyi Biotec), and Ly6C-APC (17-5932-82, eBioscience) was added for a further 10 min before washing to remove unbound antibodies. Gating thresholds were determined with unstained, singly stained, and fluorescence minus one controls. Flow cytometry (Fortessa, BD Biosciences) was performed to acquire leukocytes based on typical light scatter properties, with further gating used to define the following subsets: *1*) CD45^+^; total leukocytes; *2*) CD45^+^CD11b^+^; myeloid cells; *3*) CD45^+^CD11b^+^Ly6C^+^Ly6G^−^; monocytes; *4*) CD45^+^CD11b^+^Ly6C^+^Ly6G^+^; neutrophils; *5*) CD11b^+^Ly6C^hi−^Ly6C^−^; “inflammatory” monocytes; and *6*) CD11b^+^Ly6C^lo−^Ly6G^−^ “reparative” monocytes. All populations are expressed as cells/mL of blood (32, 33).

#### Quantification of lipid deposition in aorta and liver.

Mice fed on a Western diet for 12 wk were surgically anesthetized before terminal exsanguination by arterial perfusion via the abdominal aorta with PBS at a constant pressure of 100 mmHg with outflow through the severed jugular veins. This was followed by constant pressure perfusion in situ with 4% paraformaldehyde. The heart was removed to study the aortic sinus. In other animals, the thoracic and abdominal aorta was dissected to allow en face quantification of plaque (8, 32). To quantify lipid deposition in the liver, 5 µm thick (formalin fixed paraffin embedded sections) of the liver were cut and stained with hematoxylin and eosin. Three sections, 50 µm apart, were assessed from each liver for lipid deposition with a 330 µm^2^ region from each tissue section traced and quantified using Image-Pro Plus software (Media Cybernetics).

#### Histology of aortic sinus.

Specimens of heart were embedded in paraffin or optimal cutting temperature compound (OCT). Sections were cut at 5 µm for paraffin-embedded and 10 µm for OCT-embedded sections. Sections were cut until the aortic valve cusps were visible for the aortic sinus. Sections were stained with Miller’s elastin/van Gieson (32). Alpha-smooth muscle actin expression in sections of the aortic root was determined using a rabbit polyclonal alpha-smooth muscle actin antibody (ab5694, Abcam) and a secondary goat rabbit antibody (A11070 Alexa Fluor488, Thermofischer Scientific).

#### Elastin fragmentation.

Fragmentation of elastin was assessed by counting the number of breaks in the aortic elastin laminae at the level of the aortic sinus (22). The number of breaks was expressed per medial area, which was taken to be the area between the internal and external elastic laminae. Measurement was made using at least five serial sections per animal.

#### Analysis of antioxidant and associated gene expression in endothelial cells.

mRNA was isolated using a commercial kit (Roche), and cDNA was reverse transcribed from the RNA samples (High Capacity cDNA Reverse Transcription kit, Applied Biosystems, PN: 4368814). mRNA levels of catalase, superoxide dismutase 2 (SOD2), interleukin-1 beta (IL-1β), tumor necrosis factor-1α (TNF-α), Nox4 NADPH oxidase (Nox4), ICAM-1, VCAM-1, the C-C motif ligand 2 chemokine (CCL2), and C-C chemokine receptor type 2 (CCR2) were quantified using real-time quantitative PCR (10). Hypoxanthine-guanine phosphoribosyltransferase (HPRT) was used as an internal control in preference to GAPDH as expression of the latter is regulated by insulin (Thermo Fisher, details of the Taqman probes used are shown in Supplemental Table S4).

#### Preparation of tissue lysates, SDS-PAGE electrophoresis, and Western blotting.

Samples were homogenized in cell extraction buffer (in mmol/L, unless otherwise specified, 10 Tris pH 7.4, 100 NaCl, 20 Na_4_P_2_O_7_, 1 NaF, 2 Na_3_VO_4_, 1 EDTA, 1 EGTA, 10% glycerol, 1% Triton X-100, 0.1% SDS, and 0.5% deoxycholate) supplemented with additional protease and phosphatase inhibitors using a TissueLyser II (Qiagen). The lysate produced was centrifuged at 13,000g for 15 min at 4°C. The supernatant was removed and further diluted with an equivalent volume of cell extraction buffer before a brief sonication. All samples underwent a further centrifugation step (13,000 *g*, 10 min at 4°C) to produce a clarified lysate. Protein concentrations were determined using the Bicinchoninic acid assay (Thermo Fisher) before being resolved by SDS-PAGE electrophoresis using 4–12% polyacrylamide NuPAGE gels (Thermo Fisher) and transferred onto Immobilon-P polyvinylidine difluoride membrane (Merck Millipore). The membrane was blocked for 1 h in Tris-buffered saline containing 5% (wt/vol) bovine serum albumin (BSA; Cell Signaling) and 0.1% Tween-20 followed by incubation with primary antibodies (VCAM-1, ab174279; and ICAM-1, ab25375; Abcam) in the same buffer. Blots were incubated with the appropriate peroxidase-conjugated secondary antibodies and visualized using an enhanced chemiluminescence detection system (Merck Millipore).

#### Quantification of serum interleukin-1β activity.

Serum IL-1β activity was assessed in serum samples using a mouse IL-1β Quantikine ELISA Kit (MLB00C, R&D Systems) according to the manufacturer’s instructions.

#### Statistical methods.

The a priori selected comparison was to compare triple transgenic mice deficient in Nox2 and their double transgenic littermates with Nox2 intact and gp91dstat-treated mice with their scrambled peptide-treated littermates. Data were analyzed using unpaired Student’s *t* tests or Mann-Whitney tests where appropriate using GraphPad Prism 7.05 (*P* < 0.05 taken as statistically significant, and *n* denotes number of mice per group; unless stated otherwise, data are expressed as means ± SE).

## RESULTS

### 

#### Human saphenous vein endothelial cells from patients with diabetes are under oxidative stress due to increased generation of superoxide.

**Fig. 1.**
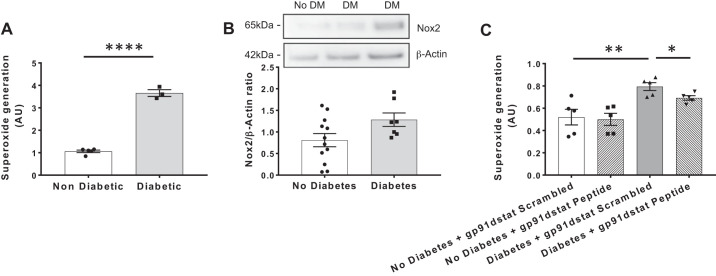
Saphenous vein endothelial cells (SVECs) from diabetic patients have increased expression of Nox2 and superoxide generation. *A*: increased NADPH-dependent superoxide generation in SVECs from patients with type 2 diabetes mellitus (No Diabetes *n* = 5; Diabetes, *n* = 3). *B*: increased expression of Nox2 protein in SVECs from patients with type 2 diabetes mellitus (No Diabetes *n* = 12; Diabetes, *n* = 7). *C*: the increased superoxide generated by SVECs from patients with type 2 diabetes is reduced by the specific Nox2 inhibitor gp91dstat. (No Diabetes *n* = 5, Diabetes *n* = 5). AU, arbitrary units; DM, diabetes mellitus. Data are expressed as mean ± SE; *n* = number of mice per genotype. *P < 0.05, **P < 0.01, ****P < 0.0001.

Experiments were performed on SVECs from a total of 25 patients (20 men, 5 women), who were divided in two groups according to diabetic status: *group 1*: no diabetes (*n* = 16), aged 68.1 ± 1.7 yr, range 59–83 yr; and *group 2*: type 2 diabetes (*n* = 9), aged 66.3 ± 3.8 yr, range 48–76 yr. All patients with diabetes mellitus were receiving oral therapy (metformin/sulfonylureas/gliptins), and one patient was also receiving insulin. Use of routine cardiovascular medications (statins, β-blockers, and antiplatelet agents) were similar in people with and without diabetes. SVECs from patients with type 2 diabetes mellitus generated more superoxide than SVECs from patients without diabetes (Fig. 1*A*). Nox2 protein expression was also higher in SVECs from patients with type 2 diabetes compared with SVECs from patients without diabetes (Fig. 1*B*). Superoxide generation from SVEC patients with type 2 diabetes was reduced by treatment with the Nox2-specific inhibitor gp91dstat. No effect was observed on SVECs from patients without diabetes (Fig. 1*C*).

#### Glucose homeostasis, lipids and arterial blood pressure in mice with endothelium-specific insulin resistance deficient in apolipoprotein E and Nox2 (ESMIRO/ApoE^−/−^/Nox2^−/y^).

**Fig. 2.**
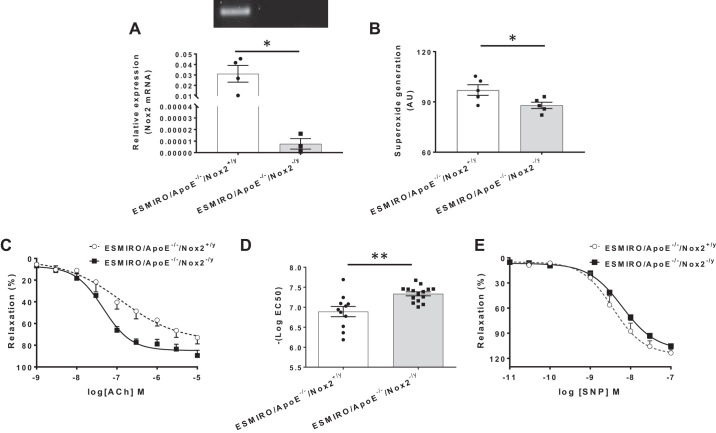
ESMIRO/ApoE^−/−^/Nox2^−/y^ have reduced endothelial cell (EC) superoxide generation and greater aortic relaxation to acetylcholine. *A*: representative image showing Nox2 mRNA was undetectable in endothelial cells from ESMIRO/ApoE^−/−^/Nox2^−/y^ mice [ESMIRO/ApoE^−/−^/Nox2^−/y^ (*n* = 3) and ESMIRO/ApoE^−/−^/Nox2^+/y^ mice (*n* = 4)]. *B*: reduced NADPH-dependent superoxide generation in endothelial cells from ESMIRO/ApoE^−/−^/Nox2^−/y^ (*n* = 5) compared with ESMIRO/ApoE^−/−^/Nox2^+/y^ mice (*n* = 5). *C*: in response to the endothelium-dependent vasorelaxant acetylcholine (Ach), aortic rings from ESMIRO/ApoE^−/−^/Nox2^−/y^ mice (*n* = 15) had greater maximal relaxation than ESMIRO/ApoE^−/−^/Nox2^+/y^ mice (*n* = 11). Both groups were fed a Western diet for 12 wk before the experiment. *D*: acetylcholine EC_50_ values derived from *C*. *E*: no difference in response to the endothelium independent vasorelaxant sodium nitroprusside (SNP) in aortic rings from ESMIRO/ApoE^−/−^/Nox2^−/y^ (*n* = 7) compared with ESMIRO/ApoE^−/−^/Nox2^+/y^ mice (*n* = 5). Both groups were fed a Western diet for 12 wk before the experiment. ESMIRO, endothelium-specific mutant insulin receptor-overexpressing mice; ApoE, apolipoprotein E; Nox2, Nox2 isoform of NADPH oxidase; AU, arbitrary units. Data are expressed as mean ± SE; *n* = number of mice per genotype. *P < 0.05, **P < 0.01.

Nox2 mRNA was undetectable in ESMIRO/ApoE^−/−^/Nox2^−/y^ mice (Fig. 2*A*). When ESMIRO/ApoE^−/−^/Nox2^−/y^ and ESMIRO/ApoE^−/−^/Nox2^+/y^ mice were compared after 12 wk on a Western diet, there was no difference in growth (Supplemental Fig. S3*A*); fasting glucose was lower in ESMIRO/ApoE^−/−^/Nox2^−/y^ *mice* (Supplemental Fig. S3*B*); and there was no difference in random serum insulin concentration (Supplemental Fig. S3*C*) or insulin tolerance testing (Supplemental Fig. S3*D*). In glucose tolerance tests, the 30-min glucose measurement was lower in ESMIRO/ApoE^−/−^/Nox2^−/y^ mice (Supplemental Fig. S3*E*). There was no difference in fasting triglycerides (Supplemental Fig. S3*F*), total cholesterol (Supplemental Fig. S3*G*), or systolic blood pressure when ESMIRO/ApoE^−/−^/Nox2^−/y^ and ESMIRO/ApoE^−/−^/Nox2^+/y^ mice were compared (Supplemental Fig. S3*H*). ESMIRO/ApoE^−/−^/Nox2^−/y^ mice had lower NADPH-dependent superoxide generation from endothelial cells compared with ESMIRO/ApoE^−/−^/Nox2^+/y^ littermates (Fig. 2*B*).

#### ESMIRO/ApoE^−/−^/Nox2^−/y^ mice have reduced endothelial dysfunction.

Aortic rings from ESMIRO/ApoE^−/−^/Nox2^−/y^ mice had greater maximal relaxation responses to acetylcholine than ESMIRO/ApoE^−/−^/Nox2^+/y^ mice (Fig. 2*C*), the significance of which was confirmed by EC_50_ values (Fig. 2*D*). There was no difference in the response to SNP when ESMIRO/ApoE^−/−^/Nox2^−/y^ and ESMIRO/ApoE^−/−^/Nox2^+/y^ mice were compared (Fig. 2*E*).

#### ESMIRO/ApoE^−/−^/Nox2^−/y^ mice have increased lipid deposition in the thoraco-abdominal aorta but no change in circulating leucocytes or proinflammatory markers in endothelial cells.

**Fig. 3.**
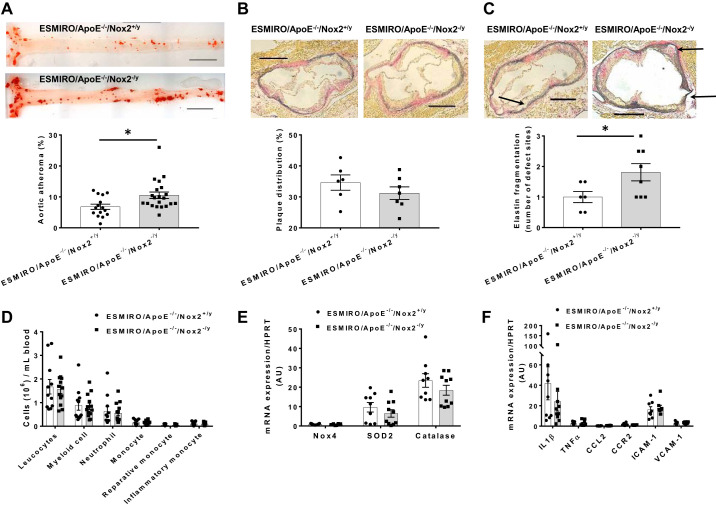
ESMIRO/ApoE^−/−^/Nox2^−/y^ mice have increased lipid deposition in the thoraco-abdominal aorta and multiple foci of elastin fragmentation. *A*: greater lipid deposition in thoraco-abdominal aorta of ESMIRO/ApoE^−/−^/Nox2^−/y^ (*n* = 21) compared with ESMIRO/ApoE^−/−^/Nox^+/y^ mice (*n* = 15). Scale bar = 250 µm. *B*: no difference in atherosclerosis at the level of the aortic sinus of ESMIRO/ApoE^−/−^/Nox2^−/y^ (*n* = 7) compared with ESMIRO/ApoE^−/−^/Nox^+/y^ mice (*n* = 6). Scale bar = 500 µm. *C*: increased elastin fragmentation in aortic wall at the level of the aortic sinus in ESMIRO/ApoE^−/−^/Nox2^−/y^ (*n* = 8) compared with ESMIRO/ApoE^−/−^/Nox^+/y^ mice (*n* = 6). Scale bar = 500 µm. *D*: similar size populations of circulating leukocytes in ESMIRO/ApoE^−/−^/Nox2^−/y^ (*n* = 13) compared with ESMIRO/ApoE^−/−^/Nox2^+/y^ mice (*n* = 11). *E*: measurement of Nox4 NADPH oxidase, superoxide dismutase 2 (SOD2) and catalase mRNA level showed no difference between ESMIRO/ApoE^−/−^/Nox2^−/y^ (*n* = 10) mice and ESMIRO/ApoE^−/−^/Nox2^+/y^ mice (*n* = 9). *F*: measurement of mRNA level showed no difference in interleukin 1β (IL-1β), tumor necrosis factor-α (TNF-α), the chemokines CCL2 and CCR2, adhesion molecules intercellular adhesion molecule 1 (ICAM-1), and vascular cell adhesion protein 1 (VCAM-1) expression in ESMIRO/ApoE^−/−^/Nox2^−/y^ (*n* = 8–10) compared with ESMIRO/ApoE^−/−^/Nox^+/y^ mice (*n* = 8–9). ESMIRO, endothelium-specific mutant insulin receptor-overexpressing mice; ApoE, apolipoprotein E; Nox2, Nox2 isoform of NADPH oxidase; AU, arbitrary units. Data are expressed as mean ± SE; *n* = number of mice per genotype. *P < 0.05.

When ESMIRO/ApoE^−/−^/Nox2^−/y^ and ESMIRO/ApoE^−/−^/Nox2^+/y^ mice were compared after 12 wk on a Western diet, ESMIRO/ApoE^−/−^/Nox2^−/y^ mice had substantially greater lipid deposition in the thoraco-abdominal aorta (Fig. 3*A*). At the level of the aortic sinus, there was no difference in atherosclerosis area (Fig. 3*B*), but ESMIRO/ApoE^−/−^/Nox2^−/y^ mice had evidence of multiple elastin fragmentations in the aortic wall, which were present in ESMIRO/ApoE^−/−^/Nox2^+/y^ mice in lower numbers (Fig. 3*C* and Supplemental Fig. S4). As vascular smooth muscle cell (VSMC) phenotypic switching has been invariably linked to atherosclerosis, we determined whether genetic ablation or pharmacological inhibition of Nox2 (using the Nox2 inhibitor gp91dstat) had any effect on VSMC phenotype by assessing the expression of heavy chain myosin (HCM). No difference in HCM expression was observed in the aortae of ESMIRO/ApoE^−/−^/Nox2^−/y^ and ESMIRO/ApoE^−/−^/Nox2^+/y^ mice or in the aortae of ESMIRO/ApoE^−/−^ mice treated with gp91dstat or scrambled peptide (Supplemental Fig. S5, *A* and *B*). Moreover, no difference in alpha-smooth muscle actin expression was observed in the aortic sinus of ESMIRO/ApoE^−/−^/Nox2^−/y^ and ESMIRO/ApoE^−/−^/Nox2^+/y^ mice (Supplemental Fig. S5*C*). To ascertain whether Nox2 genetic knockout was altering lipid deposition by affecting hepatic function, the serum levels of ALT and AST were measured in ESMIRO/ApoE^−/−^/Nox2^−/y^ and ESMIRO/ApoE^−/−^/Nox2^+/y^ mice. No differences in the levels of these enzymes were observed (Supplemental Fig. S6, *A* and *B*). In addition, no differences in hepatic lipid deposition was observed between the two groups (Supplemental Fig. S6*C*). Circulating populations of leukocytes were also similar in ESMIRO/ApoE^−/−^/Nox2^−/y^ compared with ESMIRO/ApoE^−/−^/Nox2^+/y^ mice (Fig. 3*D*). Endothelial expression of a panel of genes relevant to superoxide generation showed no difference in Nox4, catalase or SOD2 (Fig. 3*E*). There was no difference in the mRNA of a panel of proinflammatory markers (VCAM-1, ICAM-1, TNF-α, IL-1β, CCL-2, and CCR2) in endothelial cells from ESMIRO/ApoE^−/−^/Nox2^−/y^ compared with ESMIRO/ApoE^−/−^/Nox2^+/y^ mice (Fig. 3*F*).

#### Interleukin-1β activity and adhesion molecule expression.

**Fig. 4.**
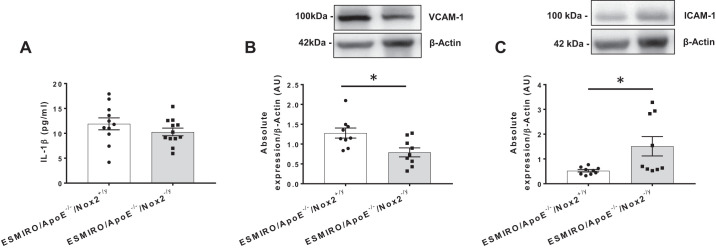
ESMIRO/ApoE^−/−^/Nox2^−/y^ mice have greater expression of ICAM-1. *A*: serum IL-1β activity was similar in ESMIRO/ApoE^−/−^/Nox2^−/y^ (*n* = 12) and ESMIRO/ApoE^−/−^/Nox2^+/y.^ mice (*n* = 11). *B* and *C*: expression of VCAM-1 was reduced (*B*) whereas expression of ICAM-1 was increased (*C*) in aorta from ESMIRO/ApoE^−/−^/Nox2^−/y^ (*n* = 9) compared with ESMIRO/ApoE^−/−^/Nox2^+/y^ mice (*n* = 9). ESMIRO, endothelium-specific mutant insulin receptor-overexpressing mice; ApoE, apolipoprotein E; Nox2, Nox2 isoform of NADPH oxidase. Data are expressed as mean ± SE; *n* = number of mice per genotype. *P < 0.05.

Serum IL-1β activity was similar in ESMIRO/ApoE^−/−^/Nox2^−/y^ and ESMIRO/ApoE^−/−^/Nox2^+/y^ mice (Fig. 4*A*). Expression of VCAM-1 was reduced (Fig. 4*B*), whereas protein expression of ICAM-1 was increased in the aorta from ESMIRO/ApoE^−/−^/Nox2^−/y^ compared with ESMIRO/ApoE^−/−^/Nox2^+/y^ mice (Fig. 4*C*).

#### Treatment with the Nox2 inhibitor gp91dstat reduces atherosclerotic progression in mice with endothelial cell-specific insulin resistance-related atherosclerosis.

**Fig. 5.**
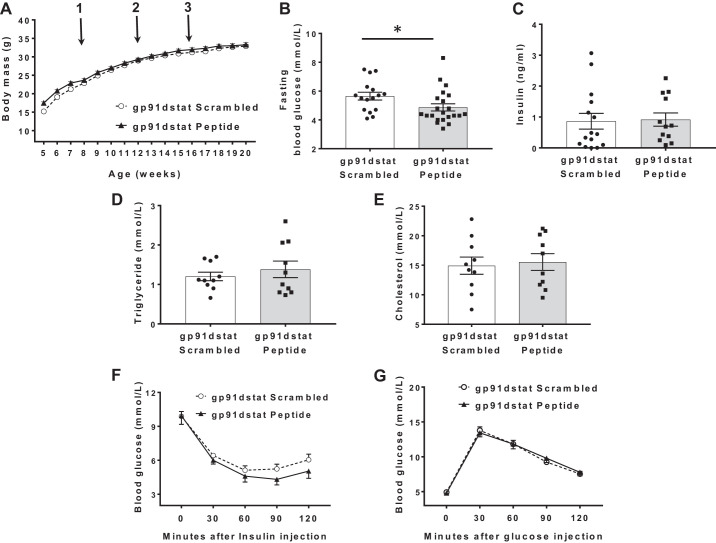
The gp91dstat treatment has no effect on growth, random insulin, fasting triglyceride, and cholesterol levels and on insulin or glucose tolerance. *A*: no difference in growth was observed between gp91dstat (*n* = 24)-treated ESMIRO/ApoE^−/−^ mice compared with mice treated with scrambled peptide (*n* = 27). *Arrow 1* denotes commencement of Western diet, and *arrows 2* and *3* denote time of minipump implantation. *B*: fasting glucose was lower in gp91dstat-treated ESMIRO/ApoE^−/−^ (*n* = 22) compared ESMIRO/ApoE^−/−^ mice treated with scrambled peptide (*n* = 16). *C*: no difference was measured in random insulin concentration in gp91dstat-treated ESMIRO/ApoE^−/−^ mice (*n* = 12) compared with ESMIRO/ApoE^−/−^ mice treated with scrambled peptide (*n* = 15). *D*: scrambled peptide-treated ESMIRO/ApoE^−/−^ mice (*n* = 10) and gp91dstat-treated ESMIRO/ApoE^−/−^ mice (*n* = 10) had similar fasting triglycerides. *E*: scrambled peptide-treated ESMIRO/ApoE^−/−^ mice (*n* = 10) and gp91dstat-treated ESMIRO/ApoE^−/−^ mice (*n* = 10) had similar fasting total cholesterol. *F* and *G*: no difference was observed in insulin (scrambled *n* = 12 vs. gp91dstat *n* = 12; *F*) or glucose tolerance tests (scrambled *n* = 19 vs. gp91dstat *n* = 19; *G*) in gp91dstat-treated ESMIRO/ApoE^−/−^ mice compared with ESMIRO/ApoE^−/−^mice treated with scrambled peptide. ESMIRO, endothelium-specific mutant insulin receptor-overexpressing mice; ApoE, apolipoprotein E. Data are expressed as mean ± SE; *n* = number of mice per genotype. *P < 0.05.

ESMIRO/ApoE^−/−^ mice treated with gp91dstat showed no difference in growth compared with those treated with scrambled peptide (Fig. 5*A*). Fasting glucose was lower (Fig. 5*B*), whereas random serum insulin concentration (Fig. 5*C*), fasting triglyceride (Fig. 5*D*), and cholesterol (Fig. 5*E*) levels were similar in gp91dstat-treated ESMIRO/ApoE^−/−^ mice compared with those treated with scrambled peptide. Insulin (Fig. 5*F*) and glucose tolerance tests (Fig. 5*G*) were also similar in gp91dstat-treated ESMIRO/ApoE^−/−^ mice compared with those treated with scrambled peptide.

**Fig. 6.**
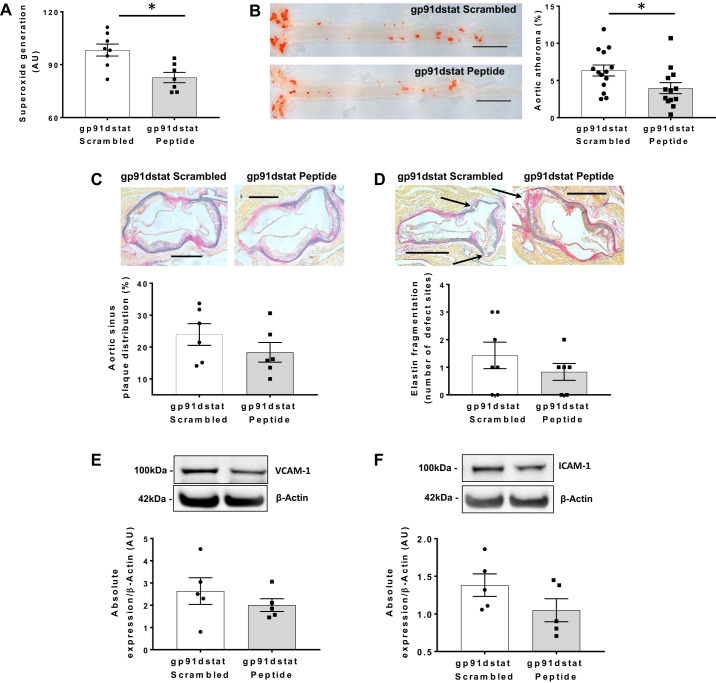
The gp91dstat treatment reduces endothelial cell (EC) superoxide generation and lipid deposition in the thoraco-abdominal aorta of ESMIRO/ApoE^−/−^ mice without causing elastin fragmentation or increased ICAM-1 expression. *A*: NADPH-dependent superoxide generation in pulmonary endothelial cells was reduced in gp91ds-tat-treated ESMIRO/ApoE^−/−^ mice (*n* = 7) compared with ESMIRO/ApoE^−/−^ mice (*n* = 8) treated with scrambled peptide. *B*: ESMIRO/ApoE^−/−^ mice treated with gp91dstat (*n* = 13) developed less atherosclerosis in the thoraco-abdominal aorta than ESMIRO/ApoE^−/−^ mice treated with scrambled peptide (*n* = 14). Scale bar = 250 µm. *C*: there was no difference in atherosclerosis at the level of the aortic sinus in gp91ds-tat-treated ESMIRO/ApoE^−/−^ mice (*n* = 6) compared with ESMIRO/ApoE^−/−^ mice (*n* = 6) treated with scrambled peptide. Scale bar = 500 µm. *D*: there was no difference in the number of defects in the aorta at the level of the sinus in ESMIRO/ApoE^−/−^ mice treated with gp91dstat (*n* = 6) compared with ESMIRO/ApoE^−/−^ mice treated with scrambled peptide (*n* = 7). Scale bar = 500 µm. *E* and *F*: VCAM-1 (*E*) and ICAM-1 (*F*) expression were not significantly different between ESMIRO/ApoE^−/−^ mice treated with gp91dstat (*n* = 5) or scrambled peptide (*n* = 5) although there was a tendency for ICAM-1 expression to be less in gp91dstat-treated mice. ESMIRO, endothelium-specific mutant insulin receptor-overexpressing mice; ApoE, apolipoprotein E; AU, arbitrary units. Data are expressed as mean ± SE; *n* = number of mice per genotype. *P < 0.05.

NADPH-dependent superoxide generation in EC was reduced in gp91dstat-treated ESMIRO/ApoE^−/−^ mice compared with mice treated with the scrambled peptide (Fig. 6*A*). ESMIRO/ApoE^−/−^ mice treated with gp91dstat developed less lipid deposition in the thoraco-abdominal aorta than mice treated with the scrambled peptide (Fig. 6*B*). However, there was no significant difference in atherosclerosis at the level of the aortic sinus (Fig. 6*C*). ESMIRO/ApoE^−/−^mice treated with gp91dstat developed a non-significant number of fewer defects in the aorta at the level of the aortic sinus than mice treated with the scrambled peptide (Fig. 6*D*). VCAM-1 and ICAM-1 expression in the aorta remained unchanged in gp91dstat-treated ESMIRO/ApoE^−/−^ mice compared with mice treated with the scrambled peptide (Fig. 6, *E* and *F*).

## DISCUSSION

Here we demonstrate that primary endothelial cells isolated from patients with type 2 diabetes and advanced atherosclerosis generate excess superoxide, the enzymatic source of which is the Nox2 isoform of NADPH oxidase. Using an in vivo model of human insulin resistance, we went on to show that complete and long-term genetic deletion of the Nox2 isoform leads to increased lipid deposition in the thoraco-abdominal aorta, substantial damage to the aortic wall, and increased expression of the adhesion molecule ICAM-1. A more conservative shorter term pharmacological approach with a Nox2-specific inhibitor reduced lipid deposition without damage to the aortic wall or increased ICAM-1 expression.

### 

#### Excess superoxide and insulin-resistant type 2 diabetes mellitus.

Excess generation of the free radical superoxide is described as oxidative stress (25). The superoxide radical is thought to promote atherosclerosis through a number of different mechanisms including, but not limited to enhanced oxidation of lipoproteins, activation of proinflammatory genes, alteration of vascular smooth muscle cell phenotype, and by reducing the bioavailability of the anti-atherosclerotic signaling radical nitric oxide (NO). Previous studies from our group have shown excess superoxide production in mice with whole body haploinsufficiency of the insulin receptor (6, 30) in mice with endothelial cell-specific insulin resistance due to expression of a dominant negative human insulin receptor (5, 30) and in mice with excessive insulin signaling in the endothelium, a model of hyperinsulinemia induced insulin resistance (32).

The source of excess superoxide generation in multiple models of insulin resistance both at the whole body level and in the endothelium has been established as the Nox2 isoform of NADPH oxidase. Studies in humans have implicated the NADPH oxidases in obesity, diabetes, and metabolic syndrome-related oxidative stress (7, 12, 28). In the present report, we identify Nox2 as the principal source of excess superoxide generation in saphenous vein endothelial cells (SVECs) taken from patients with type 2 diabetes. While SVECs are not the main players in atherosclerosis, previous studies have shown a close correlation between SVECs and arterial endothelial function demonstrating that SVECs are a good model of arterial endothelial function (13). Here we show that SVECs express increased Nox2 NADPH oxidase and generate increased superoxide, which could be inhibited by the Nox2 peptiditic inhibitor gp91dstat. This is consistent with our previous studies in mice with a range of perturbations recapitulating various aspects of insulin resistant type 2 diabetes. Hence excess generation of superoxide by Nox2 NADPH oxidase is preserved across vascular beds in mammals with insulin resistance.

Endothelial-specific mutant insulin receptor-overexpressing (ESMIRO) mice were generated to examine the contribution of endothelial cell insulin resistance on the vascular dysfunction and atherosclerosis as seen in whole body insulin resistance. These mice have endothelial cell-specific insulin resistance due to the expression of a dominant negative human insulin receptor. Our original report demonstrated that ESMIRO mice had preserved whole body glucose homeostasis but insulin resistance at the level of the endothelium, which led to excessive superoxide generation (5). In a subsequent report, we confirmed that the source of the excess superoxide in ESMIRO mice was the Nox2 isoform of NADPH oxidase (30). We went on to confirm the significance of an insulin resistant endothelium in the development of atherosclerosis by crossing the ESMIRO mouse onto an ApoE-deficient background (ESMIRO/ApoE^−/−^) to generate a model which developed accelerated atherosclerosis when compared with a mouse that was solely ApoE-deficient (8). ESMIRO mice have thus been an excellent tool to explore mechanisms of insulin resistance associated vascular oxidative stress and accelerated atherosclerosis.

#### Potential mechanisms underlying the divergent effects of pharmacological and genetic inhibition of Nox2 NADPH oxidase on insulin resistance-related atherosclerosis.

A hallmark of insulin resistance and type 2 diabetes is generation of cytotoxic concentrations of the oxidants superoxide and/or hydrogen peroxide (H_2_O_2_) and their even more toxic metabolites (2, 8). The flavoprotein Nox2 NADPH oxidase, a critical source of superoxide in insulin resistance associated oxidative stress, is expressed in endothelial cells, vascular smooth muscle cells, fibroblasts, cardiomyocytes, microglia, and phagocytic cells such as neutrophils, monocytes, and macrophages (5, 6, 27, 30, 32, 33). Although the principal effects of Nox2 activation are proinflammatory and cytotoxic (27), observations in patients with chronic granulomatous disease and preclinical models of human autoimmune disease point to a more complex role for Nox2 in inflammation and tissue damage (27). Work performed over several decades has shown that Nox2 has an important role in limiting inflammation by modulating key signaling pathways that effect neutrophil function and adaptive immunity (29).

Here we show that germline deletion of Nox2, in atherosclerosis-prone mice with endothelium-specific insulin resistance, led to increased lipid deposition in the thoraco-abdominal aorta and structural disruption of the proximal aortic wall. Consistent with our own data supporting an important role for Nox2 in maintaining the integrity of the aortic wall under disease conditions, studies have shown that germline Nox2 deficiency in atherosclerosis-prone mice accelerates the development of aortic aneurysm (15). However, a shorter term pharmacological approach to reduce Nox2 activity in atherosclerosis-prone mice with endothelium-specific insulin resistance resulted in a reduction in lipid deposition in the thoraco-abdominal aorta without disruption of the architecture of the arterial wall.

In the present study, transgenic germline knockdown or pharmacological intervention to manipulate Nox2 activity resulted in divergent effects on lipid deposition and vascular integrity of the aorta. The mechanisms underlying the different effects are likely to be complex, multifactorial, and linked to the duration of the reduced Nox2 activity. The inhibition of Nox2 activity by either of these two methods reduced superoxide generation to a similar extent suggesting that a simplistic “dose response” effect is not the reason for the distinctly different effects on the arterial wall. As discussed, Nox2 is highly expressed in granulocytes, monocytes, and macrophages and has a role in cellular and immune responses beyond its classical role in reactive oxygen species-induced microbial killing (27). Careful studies in mice with inducible cell-specific deletion of Nox2 will clarify the cell-specific role of Nox2 in insulin resistance-related atherosclerosis.

It is clear that humans and mice with chronic deficiency of Nox2 have excessive inflammation in the absence of infectious agents (16, 35). While we did not demonstrate an increase in circulating leucocytes or IL-1β [a regulator of VCAM-1 and ICAM-1 expression and a product of Nox2 inflammasome activation (36)], we were able to show that Nox2-deficient mice have reduced aortic expression of VCAM-1, which has been shown to be redox regulated (34), and increased expression of the adhesion molecule ICAM-1, a molecule thought not to be redox sensitive (19, 34). From our observations it is likely that the discrepancy in VCAM-1 and ICAM-1 is occurring as a consequence of posttranslational modification rather than transcriptional changes as we saw no changes in the mRNA expression of these adhesion molecules. Few studies have examined the effect of Nox2 deficiency on the expression of adhesion molecules in humans; however, consistent with our findings, studies in patients with chronic granulomatous disease (a genetic disorder characterized by defective NADPH oxidase activity) and colitis demonstrated increased colonic ICAM-1 expression compared with patients without chronic granulomatous disease (24). Reactive oxygen species and oxidative stress are emerging as novel players, shaping the epigenetic landscape of the entire genome (14). With accumulation evidence implicating epigenetic mechanisms in the pathophysiology of diabetes and cardiovascular disease (14), the possibility that the germline deletion of Nox2 could induce epigenetic changes [e.g., in inflammatory cells (17, 21)], which may contribute to our current observations remains intriguing.

Gray and colleagues (10) previously suggested that genetic deletion of Nox2 was potentially lethal and that the Nox1 isoform of NADPH oxidase was a more appropriate target to slow the development of atherosclerosis in diabetic mice. Consistent with this hypothesis, our study showed that a complete ablation of Nox2 NADPH oxidase led to adverse alterations in the arterial wall, whereas a potentially more conservative pharmacological approach to selectively inhibit Nox2 was advantageous despite bringing about a similar reduction in superoxide generation. However, Gray and colleagues, unlike the present report, employed a severe model of streptozotocin-induced insulin-deficient diabetes, which is more reminiscent of type 1 diabetes mellitus and itself leads to immune dysfunction (20).

In summary, we present further evidence that Nox2 is the principal enzymatic source of the excess superoxide generation from the endothelium as a hallmark of advanced type 2 diabetes. We show that complete deletion of Nox2, leads to accelerated vascular pathology in a model of human insulin resistance despite reduced superoxide generation. In contrast, we demonstrate that partial inhibition of Nox2 using a specific peptide inhibitor slows the development of aggressive vascular dysfunction.

## GRANTS

N.T.W., N.H., H.V., A.S., H.M.S., and K.J.S. are funded by British Heart Foundation Grant PG/14/54/30939.

## DISCLOSURES

No conflicts of interest, financial or otherwise, are declared by the authors.

## AUTHOR CONTRIBUTIONS

R.M.C., M.T.K., and N.Y.Y. conceived and designed research; A.M., N.T.W., N.H., H.V., A.S., N.M., A.V., H.M.S., K.B., S.K.M., K.G., K.E.P., K.J.S., P.S., A.M.S., and N.Y.Y. performed experiments; R.M.C., M.T.K., A.M., N.T.W., and N.Y.Y. analyzed data; A.M., N.T.W., R.M.C., M.T.K., and N.Y.Y. interpreted results of experiments; A.M., N.T.W., and N.Y.Y. prepared figures; A.M., N.T.W., R.M.C., M.T.K., and N.Y.Y. drafted manuscript; D.J.B., S.B.W., R.M.C., M.T.K., A.M., N.T.W., and N.Y.Y. edited and revised manuscript; A.M., N.T.W., N.H., H.V., A.S., N.M., A.V., H.M.S., K.B., S.K.M., K.G., D.J.B., S.B.W., K.E.P., K.J.S., P.S., A.M.S., R.M.C., M.T.K., and N.Y.Y. approved final version of manuscript.
